# The Landmark Series: Minimally Invasive Pancreatic Resection for Ductal Adenocarcinoma, Updates, Trends, and Future Considerations

**DOI:** 10.1245/s10434-025-17483-7

**Published:** 2025-06-07

**Authors:** Adrian Diaz, Sarah Hays, Melissa E. Hogg

**Affiliations:** 1 Department of Surgery, University of Chicago, Chicago, IL USA; 2 Center for Healthcare Outcomes and Policy, University of Michigan, Ann Arbor, MI USA; 3 Department of Surgery, NorthShore University Health System, Evanston, IL USA

## Abstract

**Background:**

Pancreatic ductal adenocarcinoma (PDAC) remains a formidable malignancy, with historically high morbidity and mortality following surgical resection. Over the past two decades, minimally invasive pancreatic resection (MIPR)—encompassing both laparoscopic and robotic approaches—has emerged as a promising alternative to conventional open techniques, offering potential advantages in perioperative recovery while aiming to maintain oncologic standards.

**Objective:**

This review aims to synthesize the evidence guiding the adoption of MIPR for PDAC and to highlight technical innovations, training considerations, and future directions in this evolving field.

**Methods:**

A focused appraisal of key retrospective analyses, prospective randomized trials, and meta-analyses was conducted. Emphasis was placed on oncologic outcomes, perioperative morbidity, learning curve dynamics, and patient selection criteria.

**Results:**

Overall, the studies reviewed suggest that MIPR can achieve equivalent oncologic outcomes—specifically in margin status and lymph node yield—relative to open resection. Advantages include reduced intraoperative blood loss, shorter hospital stays, and faster functional recovery, most consistently demonstrated for distal pancreatectomy. However, the technical complexity of minimally invasive pancreatoduodenectomy necessitates structured training and high-volume centers to mitigate safety concerns. Increasing use of neoadjuvant therapy also intersects with MIPR, necessitating careful patient selection and multidisciplinary coordination.

**Conclusions:**

Minimally invasive pancreatic resection has become a valid option for patients with PDAC, particularly for distal lesions, and holds promise for broader application pending further refinements. Rigorous training programs, thoughtful patient selection, and ongoing trials will be crucial to optimizing outcomes and solidifying MIPR as a mainstay in pancreatic cancer management.

Pancreatic ductal adenocarcinoma (PDAC) remains a formidable challenge in surgical oncology, with historically high morbidity and mortality rates associated with pancreatic resections. Over the past two decades, however, advances in surgical technology and technique have paved the way for minimally invasive pancreatic resection (MIPR), including both laparoscopic and robotic approaches. While an open approach to pancreatoduodenectomy (PD) and distal pancreatectomy (DP) has long been the gold standard, data from both retrospective studies and randomized controlled trials (RCTs) suggest that minimally invasive techniques can achieve comparable oncologic outcomes while providing potential advantages in perioperative recovery. Yet, the relative complexity of pancreatic surgery, coupled with the steep learning curve of a minimally invasive approach, has tempered the widespread adoption of these methods.

This article provides a focused review of the pivotal trials and seminal studies that have shaped current practice and continue to inform future directions in minimally invasive pancreatic surgery for PDAC. We highlight the technical developments, learning curve considerations, outcomes from key clinical trials, and emerging data regarding patient selection. We conclude with an overview of ongoing trials and potential future directions in this rapidly evolving field.

## Technical Advancements and the Learning Curve

Early attempts at laparoscopic pancreatoduodenectomy were limited by the inherent technical challenges of operating in the confined retroperitoneal space of the pancreatic head and performing complex anastomoses. Systematic reviews of minimally invasive pancreatic resections have demonstrated that although these approaches yield benefits such as reduced blood loss and shorter hospital stays, they are also characterized by steep learning curves. For example, Chan et al.^1^ highlighted that laparoscopic pancreatoduodenectomy requires structured training and high case volume to achieve proficiency. Similarly, a recent multicenter study by Lof et al.^2^ in experienced pancreatic centers highlighted that even with established expertise, the learning curve for laparoscopic distal pancreatectomy remains significant, as high as 85 cases for a textbook outcome.

Robotic-assisted surgery, by contrast, offers enhanced dexterity, three-dimensional visualization, and more ergonomic instrumentation, which may facilitate pancreatic reconstruction. In a pivotal study, Zureikat et al. demonstrated that robotic pancreatoduodenectomy (RPD) can be performed safely, with perioperative outcomes comparable to open pancreatoduodenectomy (OPD).^3^ In their series of robotic-assisted major pancreatic resections, spanning from enucleation to total pancreatectomy with auto islet transplant, the authors emphasized the versatility of the platform and reported reduced blood loss and shorter hospital stays, suggesting that the robotic platform may overcome some limitations of laparoscopic approaches in this technically demanding region of the pancreas.^4^

Further supporting these findings, Boone et al. examined the learning curve for RPD, showing that with accumulated experience, outcomes in RPD, including complication rates, operative times, and oncologic parameters, could approximate or match those of OPD.^5^ This study underscored the importance of structured training and high-volume centers in achieving optimal results. Finally, the systematic review by Chan and colleagues has detailed the learning curves for robotic pancreatoduodenectomy and robotic distal pancreatectomy, respectively, suggesting that the improved ergonomics and instrument articulation inherent to robotic systems may offer a shorter and more forgiving learning curve than traditional laparoscopic techniques.^1^ Nonetheless, regardless of the approach, these data underscore that the safe implementation of minimally invasive pancreatic surgery is contingent upon formal training and the accumulation of substantial operative experience at high-volume centers.

For complex operations, including pancreatic surgery, there is robust literature demonstrating a strong volume–outcome relationship.^6,7^ In that context, Zhang et al. performed a retrospective study in a high-volume pancreatic center, demonstrating that approximately 40 cases were required for a surgeon to achieve proficiency in robotic pancreatoduodenectomy.^8^ Beyond this threshold, operative time, blood loss, and hospital length of stay declined, supporting the notion that establishing expertise in MIPR requires a period of focused experience.

Finally, patient selection remains paramount for safely introducing MIPR. Recently, the International Study Group for Pancreatic Surgery (ISGPS) proposed a complexity and experience grading system to guide the selection of patients for minimally invasive pancreatoduodenectomy.^9^ This consensus statement includes a structured framework that correlates both the patient- and tumor-related complexity of the procedure (for example, presence of vascular involvement or the necessity of extended resection) with the experience level of the surgeon and institution. In simpler terms, a surgeon or center at the lower end of the learning curve might take on a minimally invasive distal pancreatectomy with a relatively small tumor and no vascular involvement (considered low complexity), whereas a high-volume center with extensive experience could attempt a more advanced robotic pancreatoduodenectomy requiring vascular resection (high complexity). In this way, the classification system ensures that the technical demands of each case match the capabilities of the operating team, thereby improving patient safety and outcomes. Such a tailored approach underscores the need for systematic case progression, specialized training, and careful patient selection to optimize the benefits of minimally invasive techniques.

## Synthesizing Evidence: Insights from Systematic Reviews and Meta-Analyses

Several systematic reviews and meta-analyses have provided valuable insights into the comparative effectiveness of minimally invasive and open pancreatic surgery. Peng et al. conducted a meta-analysis comparing robotic pancreatoduodenectomy (RPD) with open pancreatoduodenectomy (OPD).^10^ Their findings demonstrated that RPD was associated with significantly lower intraoperative blood loss but had longer operative times compared with OPD. Postoperatively, RPD showed no significant difference in overall complications or 30-day mortality; however, it was associated with a shorter hospital stay. Oncologic outcomes, including R0 resection rates and lymph node yields, were comparable between the two approaches, indicating that RPD maintains oncologic adequacy. This study highlighted the steep learning curve for RPD, emphasizing its feasibility primarily in high-volume centers with experienced surgeons.

Similarly, van Hilst et al. conducted a systematic review and meta-analysis evaluating oncologic outcomes of minimally invasive distal pancreatectomy (MIDP, including both laparoscopic and robotic PD) compared with open distal pancreatectomy (ODP) for pancreatic ductal adenocarcinoma (PDAC).^11^ Their study demonstrated that MIDP is oncologically equivalent to ODP. Specifically, the rates of R0 resection and lymph node yield, both critical metrics for surgical quality in PDAC, were comparable between the two approaches. In addition, MIDP was associated with lower estimated blood loss and shorter hospital stays, suggesting potential perioperative benefits over ODP. Importantly, no significant differences in overall or disease-free survival were identified between the two techniques. The authors concluded that MIDP is a safe and effective alternative to ODP in appropriately selected patients with PDAC, offering equivalent oncologic outcomes while potentially improving perioperative recovery.

## Key Clinical Trials in Minimally Invasive Pancreatic Resection

**Fig. 1:**
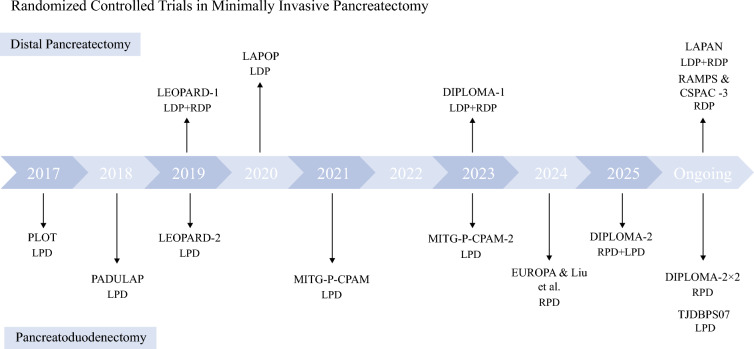
Timeline of randomized clinical trials for minimally invasive pancreatoduodenectomy and distal pancreatectomy

The evolution of minimally invasive pancreatic surgery has been shaped by a growing body of evidence from randomized controlled trials (RCTs), comparing laparoscopic and robotic approaches to traditional open techniques (Fig. 1). These trials have examined oncologic outcomes, perioperative morbidity, and overall safety, collectively shaping surgical practice and patient selection criteria. Table 1 summarizes key characteristics of completed randomized clinical trials evaluating minimally invasive pancreas resection.

**Table 1:** Summary of completed randomized clinical trials evaluating minimally invasive pancreas resection

Trial name	Citation	Location	Study design	*n*	Endpoints	Outcomes
*Pancreatoduodenectomy*						
PLOT	Palanivelu et al. 2017	India	Single-center, nonstratified, 1:1, open-label, parallel group RCT	OPD: 32 LPD: 32	**Primary:** postoperative length of hospital stay (LOS) **Secondary:** operative time, blood loss (EBL), complication rate, pathologic radicality of resection	- Shorter LOS for LPD - Longer operative time for LPD - Greater EBL in OPD - Similar rates of complications, R0 resection and number of lymph nodes retrieved
PADULAP	Poves et al. 2018	Spain	Single-center, open-label, parallel group RCT	OPD: 32 LPD: 34	**Primary**: LOS **Secondary**: operative time, blood transfusions, pancreas specific complications, postoperative complications, quality of pathologic resection	- Shorter LOS for LPD - Longer operative time for LPD - Less severe postoperative complications after LPD - No difference in transfusion requirements, pancreas-specific complications, or quality of pathologic resection
LEOPARD-2	van Hilst et al. 2019	the Netherlands	Multi-center, patient-blinded, parallel group, phase II/III RCT	OPD: 51 LPD: 54	**Primary**: functional recovery **Secondary**: Clavien–Dindo grade 3 or higher complications, pancreas-specific complications, postoperative bleeding, ICU admission, LOS, readmission, costs, quality of life	Trial was prematurely terminated by data safety and monitoring board owing to higher 90-day complication-related mortality in LPD cohort (10% versus 2% in OPD, *p* = 0.20)
MITG-P-CPAM	Wang et al. 2021	China	Multi-center, open-label, parallel group RCT	OPD: 297 LPD: 297	**Primary**: LOS **Secondary**: EBL, operative time, complication rate, mortality, Comprehensive Complication Index (CCI), survival	- Shorter LOS for LPD - Similar 90-day mortality, serious postoperative morbidities and CCI score
MITG-P-CPAM-2	Qin et al. 2024	China	3-year follow-up of MITG-P-CPAM trial	OPD: 261 LPD: 268	**Primary**: overall survival **Secondary**: long-term safety, health-related quality of life, depression	No significant difference in overall survival, quality of life, depression, or other outcomes
EUROPA	Klotz et al. 2024	Germany	Single-center, open-label, parallel group RCT	OPD: 33 RPD: 29	**Primary**: cumulative morbidity within 90 days (CCI) **Secondary**: operative time, EBL, serious adverse intraoperative events, conversion rate, quality of pathologic resection, complication rate	- Comparable CCI between RPD and OPD - Higher incidence of grade B/C pancreas-specific complications in RPD cohort - Higher rate of delayed gastric emptying in RPD cohort - Higher costs and longer operative time in the RPD cohort - No difference in EBL or 90-day mortality
–	Liu et al. 2024	China	Multi-center, 1:1, open- label RCT	OPD: 76 RPD: 78	**Primary**: LOS **Secondary**: operative time, EBL, intraoperative adverse events, blood transfusion, postoperative recovery, postoperative complications, reoperation, 90-day readmissions, 90-day mortality, pathologic findings	- Shorter LOS for RPD - Shorter operative time, lower EBL, and less transfusions for RPD - No difference in 90-day mortality rate, incidence of severe complications, or readmissions
DIPLOMA-2	Protocol: de Graaf et al. 2023 Final publication: pending	International	Multi-center, patient-blinded, 2:1 noninferiority RCT	N/A	**Primary**: CCI **Secondary**: time to functional recovery, EBL, operative time, conversion, quality of pathologic resection, clinically relevant complications, LOS, readmissions, ICU stay, survival, quality of life, cost	Final results pending publication, per abstract: -Noninferiority of RPD compared with OPD - Shorter LOS for RPD - Fast functional recovery and more daily steps for RPD - Improved perioperative outcomes, including decreased clinically relevant postoperative pancreatic fistula, EBL, and wound infections
*Distal pancreatectomy*						
LEOPARD-1	de Rooij et al. 2018	Netherlands	Multi-center, 1:1, patient-blinded RCT	ODP: 57 MIDP: 51 (5 RDP)	**Primary**: time to functional recovery after surgery **Secondary**: complications, feeding tube placement, percutaneous catheter drainage, surgical reintervention, LOS, ICU stay, readmissions, quality of life, cost	- Time to functional recovery was shorter for MIDP - Lower EBL and shorter LOS, but longer operative time for MIDP - Lower rate of delayed gastric emptying and feeding tube placement with MIDP - No difference in bleeding, surgical site infection, ICU stay, reoperation, readmission, or 90-day mortality
LAPOP	Bjornsson et al. 2020	Sweden	Single-center, unblinded, 1:1, parallel-group RCT	ODP: 29 LDP: 29	**Primary**: LOS **Secondary**: total hospital stay, readmissions, functional recovery, operative time, EBL, complications, mortality	- Shorter LOS for LDP - Shorter time to functional recovery and lower EBL with LDP - No difference in complication rates, delayed gastric emptying or clinically relevant postoperative pancreatic fistula
DIPLOMA-1	Korrel et al. 2023	International	Multi-center, patient-blinded, 1:1, noninferiority RCT	ODP: 110 MIDP: 114 (31 RDP)	**Primary**: R0 resection **Secondary**: functional recovery, LOS, overall complications, pancreas specific complications, postoperative transfusions, readmissions, mortality, survival, disease recurrence, quality of life	- MIDP was noninferior to ODP in R0 resection - Comparable rates of median lymph node yield and recurrence - Postoperative outcomes were comparable, including time to functional recovery and overall survival

*ICU* intensive care unit, *OPD* open pancreatoduodenectomy, *LPD* laparoscopic pancreatoduodenectomy, *RPD* robotic pancreatoduodenectomy, *RCT* randomized controlled trial, *LOS* length of stay, *EBL* estimated blood loss, *CCI* comprehensive complication index, *ODP* open distal pancreatectomy, *MIDP* minimally invasive distal pancreatectomy, *LDP* laparoscopic distal pancreatectomy, *RDP* robotic distal pancreatectomy

### Pancreatoduodenectomy

Two foundational studies in this area include the PLOT trial and the PADULAP trial, both of which sought to determine whether laparoscopic pancreatoduodenectomy (LPD) could offer advantages over open pancreatoduodenectomy (OPD).^12,13^ Both studies found that laparoscopic PD versus open surgery was associated with a shorter LOS and a more favorable postoperative course while maintaining oncological standards. Of note, each trial noted prolonged operative times—a tradeoff that highlights the technical demands of a laparoscopic approach. In China, the MITG-P-CPAM trial randomized patients to either undergo either LPD or OPD across 14 medical centers. The trial demonstrated shorter LOS for LPD, however, similar 90-day mortality, serious postoperative morbidities, and Comprehensive Complication Index (CCI) score.^14^ Subsequent analysis of patients with pancreatic adenocarcinoma enrolled in the trial demonstrated that laparoscopic pancreaticoduodenectomy yielded similar short-term outcomes to open pancreaticoduodenectomy, with comparable postoperative length of stay, postoperative surgical complications, number of lymph nodes harvested, and 90-day mortality.^15^ Ultimately, a 3-year follow-up of the trials demonstrated no significant difference in overall survival, quality of life, depression, or other outcomes.^16^

Following these trials, the LEOPARD-2 trial in the Netherlands, the first multi-institutional trial, sought to further assess the safety of minimally invasive pancreatoduodenectomy (MIPD). However, this trial was halted prematurely owing to safety concerns—most notably laparoscopic pancreatoduodenectomy was associated with more complication-related deaths than open pancreatoduodenectomy.^17^ While ultimately not a statistically significant difference, these findings lead to emphasis on the critical role of surgeon expertise and appropriate patient selection and ultimately the abandonment of laparoscopic pancreatoduodenectomy in the Netherlands in favor of robotics.

The steep learning curve and concerning outcomes from laparoscopic pancreatoduodenectomy, in the setting of the emergence of robotic pancreatic surgery, has prompted further investigation into its benefits over open techniques. The DIPLOMA-2 trial is an international, multicenter study designed to confirm the safety and noninferiority of MIPD compared with OPD and was presented at the International Hepato-Pancreato-Biliary Association in Cape Town South Africa in 2024.^18^ Final publication is pending; however, the abstract and presentation showed noninferiority, shorter hospital stay, faster functional recovery, more daily steps, and improved perioperative outcomes, including decreased clinically relevant postop pancreatic fistula, reduced blood loss, and decreased wound infections.

Two additional trials have demonstrated similar findings to those from the Dutch group. First, the Liu et al. study, a multicenter RCT from China, provided compelling evidence that short-term postoperative outcomes following robotic pancreatoduodenectomy (RPD) are favorable compared with OPD. Specifically, the study demonstrated that RPD leads to shorter hospital stays and fewer wound-related complications while maintaining equivalent oncologic outcomes compared with OPD.^19^ Simultaneously, the EUROPA trial, a single center trial from Germany, was the first to report on longer term outcomes from RPD. Specifically, the EUROPA trial found similar 90-day comprehensive complications and mortality rates between RPD and OPD, though the robotic arm experienced higher rates of pancreas-specific complications and longer operative times.^20^

Although these trials have corroborated the promise of robotic pancreatoduodenectomy, they have also underscored the need for specialized training to overcome learning curves and for careful patient selection when integrating robotic techniques into clinical practice. For example, the high conversion rates, such as the 23% observed in the EUROPA trial, contrasted with conversion rates less than 10% when centers surpassed the learning curve, raise important questions about whether observed differences in complication profiles and operative times are attributable to surgeon inexperience or represent inherent limitations of the technique.^21^ Moreover, international debate continues regarding the caseload required to achieve proficiency in RPD, with some commentators calling for a unified definition of the minimum number of procedures necessary to ensure optimal outcomes.^22^ These insights suggest that, beyond technical advantages, structured training programs and standardized guidelines for case volumes are critical to truly realizing the benefits of robotic pancreatoduodenectomy and ensuring patient safety. Future studies, particularly multicenter RCTs in centers that have fully surmounted the learning curve, will be essential in refining these parameters and validating the long-term advantages of minimally invasive approaches.

### Distal Pancreatectomy

Evidence supporting minimally invasive techniques in pancreatic surgery extends to distal pancreatectomy. For example, in the LEOPARD trial, a multicenter Dutch trial where patients with left-sided tumors without vascular involvement were randomized in a 1:1 ratio to MIDP (laparoscopic and robot-assisted) or ODP demonstrated that MIDP facilitated faster functional recovery and shorter hospital stays while preserving oncologic outcomes.^23^ Similarly, the LAPOP trial, a Swedish, unblinded, parallel-group, single-center, superiority trial where patients were randomized in a 1:1 ratio to LDP or ODP demonstrated that laparoscopic distal pancreatectomy was associated with shorter hospital stays and quicker recovery, reinforcing the feasibility of minimally invasive approaches in select patients.^24^ In parallel, the DIPLOMA trial, an international study, demonstrated that MIDP (laparoscopic and robot-assisted) is noninferior to ODP concerning radical resection rates and oncologic parameters, confirming the viability of laparoscopic and robotic approaches in the treatment of pancreatic cancer.^25^ Collectively, these trials underscore the viability and benefit of minimally invasive distal pancreatectomy, supporting its continued integration into surgical practice and cementing it as standard of care in appropriately selected patients.

## Future Directions and Ongoing Trials

### Pancreatoduodenectomy

As minimally invasive pancreatic surgery continues to evolve, several ongoing trials aim to refine patient selection, optimize perioperative outcomes, and establish long-term oncologic efficacy. The DIPLOMA-2 trial has a spin-off DIPLOMA 2×2 trial, which is continuing to accrue resectable pancreatic adenocarcinoma and distal cholangiocarcinoma.^26^ In this trial, patients are randomized 2:1 to RPD versus OPD. The primary outcome that will be assessed is R0 resection. In China, the PORTAL trial, a phase III noninferiority RCT conducted across multiple high-volume pancreatic and robotic surgery centers, seeks to compare robotic and open pancreatoduodenectomy in terms of time with functional recovery, postoperative morbidity, and mortality.^27^ Similarly, the TJDBPS07 trial in China is evaluating 5-year overall survival among patients undergoing laparoscopic versus open pancreatoduodenectomy, aiming to define whether minimally invasive techniques maintain oncologic integrity in the long term.^28^

### Distal Pancreatectomy

For left-sided tumors, distal pancreatectomy has also evolved through modifications such as radical antegrade modular pancreatosplenectomy (RAMPS). The RAMPS trial randomizes patients to robotic RAMPS or standard retrograde pancreatosplenectomy (SRPS) for body/tail pancreatic adenocarcinoma.^29^ Feasibility, safety, and oncologic endpoints (R0 resection rate, lymph node yield) will be examined to refine which distal approach optimizes radical resection while minimizing morbidity. Similarly, the CSPAC-3 trial is a multicenter phase III RCT comparing RAMPS versus SRPS in patients with resectable body and tail PDAC.^30^ Investigators hypothesize that RAMPS may improve oncologic outcomes (e.g., higher R0 resection rate, increased lymph node retrieval), potentially offering a new standard for left-sided pancreatic cancers. The LAPAN trial, currently underway in Japan, is evaluating whether minimally invasive distal pancreatectomy (MIDP) can achieve oncologic equivalence to an open approach.^31^

Finally, the SPLENDID study is getting off the ground and aimed at evaluating the oncological safety of spleen preservation in patients with left-sided pancreatic cancer. In this study, all patients are being treated according to standard care. After the resection of the pancreas tail and the spleen, surgeons are asked to use the back table to cut the removed specimen to the exact place where a Warshaw procedure (spleen-sparing surgery) would be performed. These preparations are sent to the pathologist to investigate how many lymph nodes are present in both sections and how many lymph node metastases are present.^32^

In summary, Table 2 summarizes key characteristics of open randomized clinical trials evaluating minimally invasive pancreas resection.

**Table 2:** Summary of open randomized clinical trials evaluating minimally invasive pancreas resection

Trial name	Location	Study design	*n*	Objective	Primary endpoint	Status
*Pancreatoduodenectomy*						
DIPLOMA-2×2	International	Multi-center, randomized, controlled, double blinded, noninferiority trial	396 patients	Compare RPD and OPD for oncological efficacy for patients with pancreatic head cancer (pancreatic ductal adenocarcinoma and distal cholangiocarcinoma)	Microscopic R0 resection rate	Currently enrolling
TJDBPS07	China	Multi-center, parallel group, open-label, noninferiority randomized controlled trial	200 patients	Compare LPD and OPD for resectable pancreatic ductal adenocarcinoma treatment	5-year overall survival rate	Currently enrolling
*Distal pancreatectomy*						
RAMPS	China	Single-center, randomized controlled trial	200 patients	Evaluate the surgical and oncological outcomes of robotic radical antegrade modular pancreatosplenectomy (RAMPS) compared with the standard retrograde pancreatosplenectomy	R0 resection margins	Currently enrolling
CSPAC-3	China	Multi-center, two-armed, blinded, intention-to-treat analysis randomized controlled trial	300 patients	Compare the effects of RAMPS and standard retrograde pancreatosplenectomy on patient survival and preoperative safety	Overall survival	Currently enrolling
LAPAN	Japan	Multi-center, randomized, phase III clinical trial	370 patients	Confirm the noninferiority of overall survival in patients with resectable pancreatic cancer undergoing MIDP compared with patients undergoing ODP	Overall survival	Currently enrolling
SPLENDID	International	Multi-center prospective observational study	N/A	To determine incidence of lymph node metastasis in the peri-splenic hilum in patients with left-sided PDAC who undergo left-sided pancreatectomy with splenectomy	Rate of lymph node metastasis in the splenic portion of the specimen	Currently enrolling

**Table 3:** Summary of recent minimally invasive pancreas resection consensus conference guidelines

Consensus conference guidelines	Miami guidelines 2020s	Brescia guidelines 2024	Paris guidelines 2025
Minimally invasive pancreatoduodenectomy	**Grade 2A**: There is insufficient data to recommend MIPD over OPD **Grade 2B**: Both MIPD and OPD are valid approaches for selected patients with adenocarcinoma **Grade 2C**: No evidence of superiority between LPD and RPD exists	**Expert Opinion**: There is insufficient evidence to define a superior anastomotic technique in either LPD or RPD, choice is surgeons preference **Grade 1C**: In MIPD, the pancreatic neck is preferentially divided from inferior to superior margin, which leads to the identification of the main pancreatic duct, which can be selectively divided with cold scissors **Grade 1C**: An artery-first approach is feasible during MIPD, and indications between MIPD and OPD are the same	**Grade 2B**: RPD performed with expertise and in selected patients is noninferior in terms of perioperative outcomes compared with OPD **Grade 2B**: RPD performed with expertise could be considered an acceptable approach for selected patients with resectable PDAC and should be considered an acceptable approach for selected patients with right-sided benign or premalignant disease
Minimally invasive distal pancreatectomy	**Grade 1B**: MIDP should be considered over ODP for benign and low-grade malignant tumors given shorter LOS, reduced EBL, and equivalent complication rates **Grade 2B**: MIDP for PDAC is safe, feasible, and oncologically efficient in experienced hands **Grade 2B**: Both LDP and RDP are safe and feasible options, decision should be made on the basis of experience and resources **Expert opinion**: No evidence regarding the use of vascular resection in MIDP	**Grade 1C:** In spleen-preserving LDP, both vessel-sparing and vessel-resecting techniques are appropriate for treatment of benign or premalignant diseases **Grade 1C**: When appropriate, the dissection between the pancreas and the splenic vessels should be carefully performed with blunt dissection and energy devices after complete mobilization of splenic flexure **Grade 1C**: The pancreatic hanging maneuver is an appropriate option during MIDP **Grade 1C:** When dividing pancreas neck, clear visualization of the splenic/portal vein junction should be obtained before ligation and division of splenic vein	**Grade 1B:** RDP should be considered an acceptable approach for patients with left-sided benign or premalignant neoplasms **Grade 2C:** RDP performed with expertise should be considered an acceptable approach for patients with resectable PDAC **Grade 2C:** RDP is associated with lower conversion and failure to preserve spleen rates than LDP
Patient selection and safety	**Grade 1C**: There are no contraindications for MIPR based on patient age, obesity, or previous abdominal surgery **Grade 1C**: No evidence exists to clearly determine the appropriate timing or indication for conversion in MIPR	**Grade 1C:** Urgent conversions are associated with adverse outcomes compared with nonurgent conversions, and effort should be made to perform elective conversion before getting into an emergency conversion	**Grade 1C**: Surgeons should decide on the most adequate surgical approach, considering training, expertise, accessibility, cost-effectiveness, and patient-specific factors **Grade 2C/D**: Patient age or BMI should not be considered an absolute contraindication for the robotic approach, nor should previous abdominal surgery
Experience and Training	**Grade 1B**: Center volume strongly affects the outcomes after MIPR. MIPD should be performed in high- volume centers since morbidity and mortality are worse when performed in low-volume centers **Grade 1C:** The value of formal MIPR training is safe introduction and expansion of the technique. Participation in a structured training program is strongly recommended	**Grade 1B**: Center volume strongly affects outcomes after LPD and RPD; morbidity, mortality, and R0 rates are better when performed in centers with > 20 LPD or RPD per year **Grade 1B**: The learning curve for operative time is 16 procedures for LDP and 39 procedures for LPD (longer for postoperative complications). The learning curve for operative time is 15 procedures for RDP and 25 procedures for RPD (longer for postoperative complications) **Grade 2B**: Formal mentorship and structured training programs facilitate the safe introduction and expansion of LPR and RPR	**Grade 1B**: A standardized robotic training program is necessary and should be tailored to the procedure and robotic surgical system and follow stepwise proficiency-based curricula **Grade 1C**: A structured process, including a standardized training program, should exist at a center level for credentialing, privileging, and embarking surgeons on an HPB robotic program

*BMI* body mass index, *HPB* hepato-pancreato-biliary

It should be noted that clinical trials evaluating minimally invasive approaches to pancreas surgery are extremely challenging for various reasons, including the steep learning curve, variability in surgeon expertise, and difficulties in standardizing techniques across institutions. Specifically, when assessing the value of a new surgical intervention, finding the optimal timing for such a study can be elusive—when performed too early, the intervention effects may be contaminated by the learning curve—if performed too late, surgeons and/or patients may feel that “equipoise” has been lost and may not be interested to join the trial. For example, the EUROPA trial saw 12% of patients declining participation, while 21% did so in the Chinese trial.^19,20^ Further, it is noteworthy that the US-based RCT NCT04171440 could not be completed owing to difficulties in recruiting patients for OPD and was “converted” into an observational study on perioperative outcomes of RPD.^33^

## Trends and Future Considerations

**Fig. 2:**
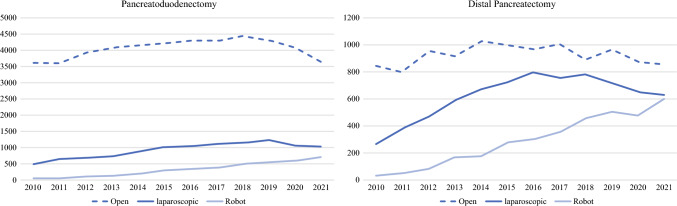
Trends in surgical approaches for pancreatoduodenectomy and distal pancreatectomy, 2010–2021

Over the past two decades, minimally invasive pancreatic resection (MIPR) has evolved dramatically, fueled by advances in technology, increasing surgeon expertise, and a robust body of evidence supporting its efficacy and safety (Fig. 2). While minimally invasive pancreatoduodenectomy (MIPD) holds significant promise, its technical complexity necessitates a cautious approach for programs embarking on its adoption. Evidence continues to support that centers initiating MIPD should ensure that they possess not only the requisite expertise and volume, but also a structured training framework to overcome the steep learning curve and mitigate potential risks. In contrast, minimally invasive distal pancreatectomy (MIDP) has emerged as the standard of care, demonstrating superior perioperative outcomes and faster recovery compared with traditional open techniques. Now, in order to have an accredited Hepatico-Pancreatico-Biliary fellowship, the Americas Hepatico-Pancreatico-Biliary Association requires 5 MIDP cases for graduation, affirming the importance of this approach. Furthermore, given the high level of evidence for MIDP, several recent international consensus conference guidelines held across the world, have advocated for MIDP (Table 3). The national trends depicted from the National Cancer Database show stagnation of ODP with the combined rise of LDP and RDP, showing MIDP has surpassed ODP. Given this, these authors advocate MIDP is the current standard of care.

Furthermore, technological advancements are significantly shaping the future of minimally invasive pancreatic resection. For example, a recent review has identified at least 20 new and upcoming robotic platforms, underscoring the rapid innovation and competition occurring in this space.^34^ Robotic platforms can be broadly categorized into standalone systems and laparoscopic adjunct systems. Standalone robotic systems, such as Medtronic’s Hugo Robotic-Assisted Surgery System (USA) and CMR Surgical’s Versius (USA), offer fully modular and portable designs that aim to directly compete with the traditional Intuitive da Vinci platform. These standalone systems typically feature multiple independent robotic arms—such as Versius's four-arm configuration with 360° wrist rotation capabilities and Hugo’s modular multi-cart architecture—designed to enhance surgeon ergonomics, operational flexibility, and potentially reduce costs through innovative pricing models.^34,35^ Additionally, Johnson & Johnson's Ottava platform integrates four robotic arms directly into the surgical table, providing a unified design that optimizes space and movement efficiency. The platform's twin motion feature uniquely allows patient repositioning without interrupting surgery, further enhancing intraoperative flexibility and workflow efficiency.

In contrast, laparoscopic adjunct systems like Distal Motion’s Dexter (Switzerland) serve as hybrid platforms, integrating seamlessly with existing laparoscopic equipment. Dexter facilitates swift transitions between laparoscopic and robotic techniques without needing full undocking, emphasizing its suitability for outpatient settings with simplified setup. Additionally, robotic surgical assistants like Moon Surgical's Maestro (USA) offer two-arm units that act primarily as camera holders and retractors, complementing conventional laparoscopic surgery. These systems promise future enhancements through artificial intelligence capabilities, such as automatic camera tracking of instruments, further improving surgical precision and ease of use.^34^

Emerging single-port robotic systems represent another innovative approach, aiming to significantly reduce invasiveness by operating through a single small incision. Virtual Incision's MIRA (USA) platform, fully housed within the patient's abdominal cavity, provides internal triangulation with highly compact instrumentation, while Vicarious (USA) Surgical’s system features advanced multi-articulating robotic arms that enable intricate procedures through a single port.^34^ These platforms have the potential to further revolutionize minimally invasive pancreatic resections, offering surgeons the ability to perform complex procedures with reduced patient trauma and potentially quicker recoveries.

Artificial intelligence (AI) is also being explored for robotic assistance, with emerging prototypes demonstrating autonomous suturing capabilities, hinting at a future where specific tasks in pancreatic surgery may be partially automated.^36^ These systems integrate advanced features such as tremor filtration, motion scaling, and near-infrared imaging, enhancing surgical precision. Further innovation in robotic stapling and cutting tools, as well as the integration of real-time imaging overlays, is expected to refine robotic capabilities and broaden their clinical applications.^34^

Artificial intelligence is also being leveraged to improve preoperative planning, intraoperative guidance, and postoperative outcome prediction. AI-assisted imaging analysis enhances surgical planning by generating 3D models that help map tumors, vasculature, and adjacent structures, reducing complications and optimizing patient selection.^37,38^ For example, 3D visualization and virtual simulation of pancreatic anatomy can help surgeons navigate the complex relationship of tumors to vessels. Studies show that patients who underwent liver or pancreatic surgery with the aid of 3D operative planning had shorter operative times and fewer complications than those planned with standard imaging.^38^ In pancreatic cancer, AI-driven radiomic analysis of CT/MRI scans can identify subtle imaging features correlated with tumor aggressiveness or fibrosis, improving the assessment of resectability and likely margins.^39^ Furthermore, machine learning algorithms have also been used to predict which patients may benefit from surgery; for instance, models combining radiologic and clinical data can estimate the probability of early recurrence or metastasis, informing the decision to proceed to surgery versus neoadjuvant therapy.^40^

Intraoperatively, AI-driven augmented reality (AR) overlays enhance navigation, providing real-time visual guidance for tumor resection and vascular dissection. The Augmented Reality Assistance System (ARAS), for example, has been shown to reduce operative time, blood loss, and positive surgical margins.^41^ Imaging overlays, including fluorescence-guided surgery (FGS) and AR, are further improving intraoperative visualization. Near-infrared (NIR) fluorescence imaging with indocyanine green (ICG) is widely used to assess bile duct anatomy, vascular perfusion, and anastomotic integrity. While consensus supports its utility as an adjunct tool, the development of tumor-specific fluorescent probes remains an area of active research. Future integration of AR with fluorescence imaging could allow real-time visualization of tumor margins and vascular structures within the surgical field, enhancing precision and safety.^42^ As these technologies continue to advance, establishing standardized protocols for their implementation and evaluating their impact on long-term oncologic outcomes will be crucial.

The continued integration of robotics, AI, and imaging technologies into minimally invasive pancreatic surgery requires structured training programs and careful patient selection to ensure safe implementation. Prospective studies and multicenter trials are essential to validate their benefits, refine best practices, and optimize cost-effectiveness. As these innovations mature, they have the potential to enhance surgical precision, improve perioperative outcomes, and establish minimally invasive techniques as the standard of care for pancreatic cancer treatment.

Despite these innovations, implementation challenges persist. The steep learning curve required for mastering minimally invasive pancreatic techniques remains a substantial barrier. High-quality training programs, simulation-based learning, and mentorship at high-volume centers are essential to overcoming this obstacle. Additionally, disparities in access to robotic platforms and advanced laparoscopic technologies limit the widespread adoption of MIPR, necessitating the development of cost-effective solutions and equitable resource distribution. Future research directions should prioritize long-term oncologic outcomes, cost-effectiveness, and recurrence patterns associated with minimally invasive approaches. Expanding large-scale RCTs and multicenter registries will provide critical data to refine surgical techniques and ensure the safe and effective integration of MIPR into standard pancreatic cancer treatment algorithms.

## Conclusion

Minimally invasive pancreatic resection represents a paradigm shift in pancreatic cancer surgery, offering potential advantages in perioperative recovery while maintaining oncologic rigor. However, its widespread adoption is contingent on meticulous patient selection, rigorous surgeon training, and continued clinical investigation. While early data suggest oncologic equivalence to open surgery in well-selected patients, ongoing trials will further define the role of MIPR in PDAC treatment.

The road ahead demands a multidisciplinary effort to address technical, educational, and accessibility challenges. Expanding access to structured training programs, fostering innovation in robotic and laparoscopic platforms, and refining neoadjuvant treatment strategies will be key to ensuring the safe and effective implementation of MIPR. As evidence continues to evolve, future research must focus on refining surgical techniques, evaluating long-term outcomes, and addressing barriers to accessibility, ensuring that the benefits of minimally invasive pancreatic surgery reach a broader patient population. By doing so, we move closer to establishing MIPR as a standard of care, improving both survival and quality of life for patients with pancreatic cancer.

## References

[1] Chan KS, Wang ZK, Syn N, Goh BKP. Learning curve of laparoscopic and robotic pancreas resections: a systematic review. *Surgery*. 2021;170(1):194–206. 10.1016/j.surg.2020.11.046. 33541746 10.1016/j.surg.2020.11.046

[2] Lof S, Claassen L, Hannink G, et al. Learning curves of minimally invasive distal pancreatectomy in experienced pancreatic centers. *JAMA Surg*. 2023;158(9):927–33. 10.1001/jamasurg.2023.2279. 37378968 10.1001/jamasurg.2023.2279 PMC10308297

[3] Zureikat AH, Nguyen KT, Bartlett DL, Zeh HJ, Moser AJ. Robotic-assisted major pancreatic resection and reconstruction. *Arch Surg Chic Ill*. 2011;146(3):256–61. 10.1001/archsurg.2010.246. 10.1001/archsurg.2010.246 21079111

[4] Zureikat AH, Moser AJ, Boone BA, Bartlett DL, Zenati M, Zeh HJ. 250 Robotic pancreatic resections: safety and feasibility. *Ann Surg*. 2013;258(4):554–62. 10.1097/SLA.0b013e3182a4e87c. 24002300 10.1097/SLA.0b013e3182a4e87c PMC4619895

[5] Boone BA, Zenati M, Hogg ME, et al. Assessment of quality outcomes for robotic pancreaticoduodenectomy: identification of the learning curve. *JAMA Surg*. 2015;150(5):416–22. 10.1001/jamasurg.2015.17. 25761143 10.1001/jamasurg.2015.17

[6] Birkmeyer JD, Siewers AE, Finlayson EVA, et al. Hospital volume and surgical mortality in the United States. *N Engl J Med*. 2002;346(15):1128–37. 10.1056/NEJMsa012337. 11948273 10.1056/NEJMsa012337

[7] Birkmeyer JD, Stukel TA, Siewers AE, Goodney PP, Wennberg DE, Lucas FL. Surgeon volume and operative mortality in the United States. *N Engl J Med*. 2003;349(22):2117–27. 10.1056/NEJMsa035205. 14645640 10.1056/NEJMsa035205

[8] Zhang T, Zhao ZM, Gao YX, Lau WY, Liu R. The learning curve for a surgeon in robot-assisted laparoscopic pancreaticoduodenectomy: a retrospective study in a high-volume pancreatic center. *Surg Endosc*. 2019;33(9):2927–33. 10.1007/s00464-018-6595-0. 30483970 10.1007/s00464-018-6595-0

[9] Barreto SG, Strobel O, Salvia R, et al. Complexity and experience grading to guide patient selection for minimally invasive pancreatoduodenectomy: an ISGPS consensus. *Ann Surg*. Published online 22 July 2024. 10.1097/SLA.0000000000006454 10.1097/SLA.0000000000006454 39034920

[10] Peng L, Lin S, Li Y, Xiao W. Systematic review and meta-analysis of robotic versus open pancreaticoduodenectomy. *Surg Endosc*. 2017;31(8):3085–97. 10.1007/s00464-016-5371-2. 27928665 10.1007/s00464-016-5371-2

[11] van Hilst J, Korrel M, de Rooij T, et al. Oncologic outcomes of minimally invasive versus open distal pancreatectomy for pancreatic ductal adenocarcinoma: a systematic review and meta-analysis. *Eur J Surg Oncol J Eur Soc Surg Oncol Br Assoc Surg Oncol*. 2019;45(5):719–27. 10.1016/j.ejso.2018.12.003. 10.1016/j.ejso.2018.12.003 30579652

[12] Palanivelu C, Senthilnathan P, Sabnis SC, et al. Randomized clinical trial of laparoscopic versus open pancreatoduodenectomy for periampullary tumours. *Br J Surg*. 2017;104(11):1443–50. 10.1002/bjs.10662. 28895142 10.1002/bjs.10662

[13] Poves I, Burdío F, Morató O, et al. Comparison of perioperative outcomes between laparoscopic and open approach for pancreatoduodenectomy: the PADULAP randomized controlled trial. *Ann Surg*. 2018;268(5):731–9. 10.1097/SLA.0000000000002893. 30138162 10.1097/SLA.0000000000002893

[14] Wang M, Li D, Chen R, et al. Laparoscopic versus open pancreatoduodenectomy for pancreatic or periampullary tumours: a multicentre, open-label, randomised controlled trial. *Lancet Gastroenterol Hepatol*. 2021;6(6):438–47. 10.1016/S2468-1253(21)00054-6. 33915091 10.1016/S2468-1253(21)00054-6

[15] Wang M, Pan S, Qin T, et al. Short-term outcomes following laparoscopic vs open pancreaticoduodenectomy in patients with pancreatic ductal adenocarcinoma: a randomized clinical trial. *JAMA Surg*. 2023;158(12):1245–53. 10.1001/jamasurg.2023.5210. 37878305 10.1001/jamasurg.2023.5210 PMC10600717

[16] Qin T, Zhang H, Pan S, et al. Effect of laparoscopic and open pancreaticoduodenectomy for pancreatic or periampullary tumors. *Ann Surg*. 2024;279(4):605–12. 10.1097/SLA.0000000000006149. 37965767 10.1097/SLA.0000000000006149 PMC10922659

[17] van Hilst J, de Rooij T, Bosscha K, et al. Laparoscopic versus open pancreatoduodenectomy for pancreatic or periampullary tumours (LEOPARD-2): a multicentre, patient-blinded, randomised controlled phase 2/3 trial. *Lancet Gastroenterol Hepatol*. 2019;4(3):199–207. 10.1016/S2468-1253(19)30004-4. 30685489 10.1016/S2468-1253(19)30004-4

[18] de Graaf N, Emmen AMLH, Ramera M, et al. Minimally invasive versus open pancreatoduodenectomy for pancreatic and peri-ampullary neoplasm (DIPLOMA-2): study protocol for an international multicenter patient-blinded randomized controlled trial. *Trials*. 2023;24(1):665. 10.1186/s13063-023-07657-7. 37828593 10.1186/s13063-023-07657-7 PMC10571285

[19] Liu Q, Li M, Gao Y, et al. Effect of robotic versus open pancreaticoduodenectomy on postoperative length of hospital stay and complications for pancreatic head or periampullary tumours: a multicentre, open-label randomised controlled trial. *Lancet Gastroenterol Hepatol*. 2024;9(5):428–37. 10.1016/S2468-1253(24)00005-0. 38428441 10.1016/S2468-1253(24)00005-0

[20] Klotz R, Mihaljevic AL, Kulu Y, et al. Robotic versus open partial pancreatoduodenectomy (EUROPA): a randomised controlled stage 2b trial. *Lancet Reg Health Eur*. 2024;39:100864. 10.1016/j.lanepe.2024.100864. 38420108 10.1016/j.lanepe.2024.100864 PMC10899052

[21] De Graaf N, Hilal MA, Besselink MG. Robotic pancreatoduodenectomy: an ongoing exploration. *Lancet Reg Health - Eur*. 2024;39:100880. 10.1016/j.lanepe.2024.100880. 38464481 10.1016/j.lanepe.2024.100880 PMC10924121

[22] Bannone E, Marchegiani G. Robotic pancreatoduodenectomy: preparing for the future. *Lancet Gastroenterol Hepatol*. 2024;9(5):395–7. 10.1016/S2468-1253(24)00036-0. 38428440 10.1016/S2468-1253(24)00036-0

[23] de Rooij T, van Hilst J, van Santvoort H, et al. Minimally invasive versus open distal pancreatectomy (LEOPARD): a multicenter patient-blinded randomized controlled trial. *Ann Surg*. 2019;269(1):2–9. 10.1097/SLA.0000000000002979. 30080726 10.1097/SLA.0000000000002979

[24] Björnsson B, Larsson AL, Hjalmarsson C, Gasslander T, Sandström P. Comparison of the duration of hospital stay after laparoscopic or open distal pancreatectomy: randomized controlled trial. *Br J Surg*. 2020;107(10):1281–8. 10.1002/bjs.11554. 32259297 10.1002/bjs.11554

[25] Korrel M, Jones LR, van Hilst J, et al. Minimally invasive versus open distal pancreatectomy for resectable pancreatic cancer (DIPLOMA): an international randomised non-inferiority trial. *Lancet Reg Health Eur*. 2023;31:100673. 10.1016/j.lanepe.2023.100673. 37457332 10.1016/j.lanepe.2023.100673 PMC10339208

[26] E-MIPS REGISTRY | DIPLOMA 2X2. Accessed 10 February 2025. https://www.e-mips.com/diploma-2x2-trial

[27] Jin J, Shi Y, Chen M, et al. Robotic versus open pancreatoduodenectomy for pancreatic and periampullary tumors (PORTAL): a study protocol for a multicenter phase III non-inferiority randomized controlled trial. *Trials*. 2021;22(1):954. 10.1186/s13063-021-05939-6. 34961558 10.1186/s13063-021-05939-6 PMC8711152

[28] Pan S, Qin T, Yin T, et al. Laparoscopic versus open pancreaticoduodenectomy for pancreatic ductal adenocarcinoma: study protocol for a multicentre randomised controlled trial. *BMJ Open*. 2022;12(4):e057128. 10.1136/bmjopen-2021-057128. 10.1136/bmjopen-2021-057128 PMC8981294 35379633

[29] Zhang G, Kang Y, Zhang H, Wang F, Liu R. Robotic radical antegrade modular pancreatosplenectomy (RAMPS) versus standard retrograde pancreatosplenectomy (SRPS): study protocol for a randomized controlled trial. *Trials*. 2020;21(1):306. 10.1186/s13063-020-04250-0. 32245518 10.1186/s13063-020-04250-0 PMC7119168

[30] Li J, Shi S, Liu J, et al. Radical antegrade modular pancreatosplenectomy (RAMPS) versus standard retrograde pancreatosplenectomy (SRPS) for resectable body and tail pancreatic adenocarcinoma: protocol of a multicenter, prospective, randomized phase III control trial (CSPAC-3). *Trials*. 2023;24(1):541. 10.1186/s13063-023-07456-0. 37592267 10.1186/s13063-023-07456-0 PMC10436627

[31] Ikenaga N, Hashimoto T, Mizusawa J, et al. A multi-institutional randomized phase III study comparing minimally invasive distal pancreatectomy versus open distal pancreatectomy for pancreatic cancer; Japan Clinical Oncology Group study JCOG2202 (LAPAN study). *BMC Cancer*. 2024;24(1):231. 10.1186/s12885-024-11957-9. 38373949 10.1186/s12885-024-11957-9 PMC10875854

[32] SPLENDID study, all elective pancreatic tail resections at PDAC | DPCG. Accessed 10 February 2025. https://dpcg.nl/studie/splendid-studie/

[33] Perioperative outcomes of robotic approach for pancreaticoduodenectomy: a multi-center, prospective, single arm, observational study. clinicaltrials.gov; 2025. Accessed 14 February 2025. https://clinicaltrials.gov/study/NCT04171440

[34] Sarin A, Samreen S, Moffett JM, et al. Upcoming multi-visceral robotic surgery systems: a SAGES review. *Surg Endosc*. 2024;38(12):6987–7010. 10.1007/s00464-024-11384-8. 39542888 10.1007/s00464-024-11384-8 PMC11615118

[35] Marchegiani F, Siragusa L, Zadoroznyj A, et al. New robotic platforms in general surgery: what’s the current clinical scenario? *Medicina (Mex).* 2023;59(7):1264. 10.3390/medicina59071264 10.3390/medicina59071264 PMC10386395 37512075

[36] Saeidi H, Opfermann JD, Kam M, et al. Autonomous robotic laparoscopic surgery for intestinal anastomosis. *Sci Robot.* 2022;7(62):eabj2908. 10.1126/scirobotics.abj2908 10.1126/scirobotics.abj2908 PMC8992572 35080901

[37] Cheng H, Xu H, Peng B, et al. Illuminating the future of precision cancer surgery with fluorescence imaging and artificial intelligence convergence. *NPJ Precis Oncol*. 2024;8(1):196. 10.1038/s41698-024-00699-3. 39251820 10.1038/s41698-024-00699-3 PMC11385925

[38] Bari H, Wadhwani S, Dasari BVM. Role of artificial intelligence in hepatobiliary and pancreatic surgery. *World J Gastrointest Surg*. 2021;13(1):7–18. 10.4240/wjgs.v13.i1.7 10.4240/wjgs.v13.i1.7 PMC7830072 33552391

[39] Marti-Bonmati L, Cerdá-Alberich L, Pérez-Girbés A, et al. Pancreatic cancer, radiomics and artificial intelligence. *Br J Radiol*. 2022;95(1137):20220072. 10.1259/bjr.20220072 10.1259/bjr.20220072 PMC10996946 35687700

[40] Koh DM, Papanikolaou N, Bick U, et al. Artificial intelligence and machine learning in cancer imaging. *Commun Med.* 2022;2(1):1–14. 10.1038/s43856-022-00199-0 10.1038/s43856-022-00199-0 PMC9613681 36310650

[41] Javaheri H, Ghamarnejad O, Widyaningsih R, et al. Enhancing Perioperative Outcomes of Pancreatic Surgery with Wearable Augmented Reality Assistance System: A Matched-Pair Analysis. *Ann Surg Open.* 2024;5(4):e516. 10.1097/AS9.0000000000000516 10.1097/AS9.0000000000000516 PMC11661739 39711676

[42] de Muynck LDAN, White KP, Alseidi A, et al. Consensus statement on the use of near-infrared fluorescence imaging during pancreatic cancer surgery based on a delphi study: surgeons’ perspectives on current use and future recommendations. *Cancers*. 2023;15(3):652. 10.3390/cancers15030652 10.3390/cancers15030652 PMC9913161 36765609

